# Improving cardiac cine MRI on 3T using 2D k-t accelerated auto-calibrating parallel imaging

**DOI:** 10.1186/1532-429X-16-S1-W3

**Published:** 2014-01-16

**Authors:** Peng Lai, Anja Brau

**Affiliations:** 1 MR Applications and Workflow, GE Healthcare, Menlo Park, California, USA; 2 MR Applications and Workflow, GE Healthcare, Garching, Munchen, Germany

## Background

Recently, 3D cine has gained attention due to its capability for single breath-hold volumetric measurement [1]. Nonetheless, conventional 2D cine maintains two intrinsic advantages, namely, its superior blood-myocardium contrast owing to blood in-flow effects and reduced SSFP banding artifacts with more localized slice-by-slice shimming. These advantages are more prominent at high field. This work intends to optimize a k-t acceleration method, kat ARC [2], for 2D cine and preliminarily investigate its performance vs. 3D cine at 3T.

## Methods

**Figure 1:**
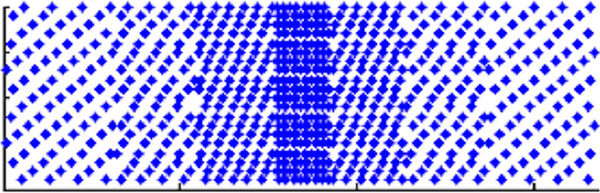
**VDkt sampling pattern for 6x outer & 2x center net acceleration**. (x: phase encoding, y: cardiac phases).

Variable-Density k-t Sampling (VDkt): As shown in Figure 1, the entire k-t space is divided into sub-bands based on distance from central k-space and these sub-bands are sampled using time-shifted acquisition with linearly increasing acceleration from center to outer bands. Static Tissue Removal (STR): Signals of static tissue voxels can be estimated from the original undersampled k-t data [3]. Such static tissue signals can be subtracted from k-space such that the subsequent k-t reconstruction processes only dynamic tissue voxels within a smaller FOV and thus produces higher image quality [3]. The same method can be used to improve k-t accelerated 2D cine, especially at apical slices where dynamic signals originates from a very small heart transection. Short-axis 2D cine SSFP data was acquired on 3 volunteers on a GE 3T with a 32-channel cardiac coil. Imaging parameters were: 360 × 270 mm2 FOV, 224 × 168 matrix, 15 8 mm slices, 40° flip angle, 8 breathholds. Full k-space was collected and offline downsampled to simulate k-t sampling with various accelerations: A. 5x outer & 1x center (net: 3.6x), B. 6x outer & 2x center (net: 5.0x); C. 7x outer & 3x center (net: 6.2x). For comparison, breath-held (15 s) 3D cine was also performed with 40° flip angle and 9x center & 3x outer acceleration (net 9.6x).

## Results

**Figure 2:**
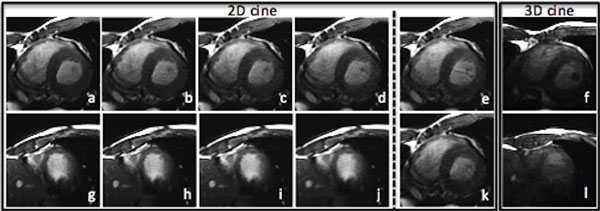
**Late systolic mid-ventricular slice using full k-space (a) and VDkt sampling with 3.6x (b), 5.0x (c) & 6.2x (d)**. Early-diastolic apical slice using full k-space (g) and VDkt with 3.6x (h), 5.0x (i) & 6.2x(j). (e) & (k) are 6.2x images using regular k-t sampling and VDkt without STR. (f) & (l) are 3D cine images with 9.6x.

As shown in Figure 2, VDkt (d) reduces aliasing artifacts vs. regular k-t sampling (e) with same acceleration and STR suppresses residual artifacts (d vs. k). At mid-ventricular slice, VDkt 5.0x (c) provides image quality similar to full k-space reference (a). At apical slice, higher acceleration of 6.2x (j) is obtainable without visible aliasing or blurring compared to full k-space image (g). In comparison, 3D cine (f, l) suffers from much lower contrast, especially near apex, and more severe banding artifacts at inferoposterior endocardium.

## Conclusions

In conclusion, the proposed approach is promising for highly accelerated 2D cine MRI. The 2D cine approach provides improved contrast and robustness vs. 3D cine and thus may be more reliable on 3T. 2D acquisition further enables flexible slice-specific selection of acceleration based on the extent of signal dynamics on each slice. Based on our results, the projected 2D cine scan time can be reduced to ~2 s/slice and totally two 16 s breath-holds for 16 slices - a reduction of 4-5x vs. full acquisition
